# The Electrical Activity of the Temporal and Masseter Muscles in Patients with TMD and Unilateral Posterior Crossbite

**DOI:** 10.1155/2015/259372

**Published:** 2015-03-26

**Authors:** Krzysztof Woźniak, Liliana Szyszka-Sommerfeld, Damian Lichota

**Affiliations:** ^1^Department of Orthodontics, Pomeranian Medical University of Szczecin, Al. Powst. Wlkp. 72, 70111 Szczecin, Poland; ^2^Department of Conservative Dentistry, Pomeranian Medical University of Szczecin, 70111 Szczecin, Poland

## Abstract

The aim of this study was to assess the influence of unilateral posterior crossbite on the electrical activity of the temporal and masseter muscles in patients with subjective symptoms of temporomandibular dysfunctions (TMD). The sample consisted of 50 patients (22 female and 28 male) aged 18.4 to 26.3 years (mean 20.84, SD 1.14) with subjective symptoms of TMD and unilateral posterior crossbite malocclusion and 100 patients without subjective symptoms of TMD and malocclusion (54 female and 46 male) aged between 18.4 and 28.7 years (mean 21.42, SD 1.06). The anamnestic interviews were conducted according to a three-point anamnestic index of temporomandibular dysfunction (Ai). Electromyographical (EMG) recordings were performed using a DAB-Bluetooth Instrument (Zebris Medical GmbH, Germany). Recordings were carried out in the mandibular rest position and during maximum voluntary contraction (MVC). Analysis of the results of the EMG recordings confirmed the influence of unilateral posterior crossbite on variations in spontaneous muscle activity in the mandibular rest position and maximum voluntary contraction. In addition, there was a significant increase in the Asymmetry Index (As) and Torque Coefficient (Tc), responsible for a laterodeviating effect on the mandible caused by unbalanced right and left masseter and temporal muscles.

## 1. Introduction

Bilateral symmetry, characteristic of vertebrates, is extremely important for describing the morphology of the masticatory organ. This feature is strongly marked in the craniofacial area and is an important determinant of its correct structure. Assessment of bilateral symmetry in the craniofacial area is a fundamental component of the examination and description of people with and without disorders. It must be stated, however, that slight facial asymmetry is acceptable, being a common and frequently observed morphological feature [1, 2]. Such a disruption of symmetry is not a significant exception but a commonly accepted structural deviation. Unfortunately, the extent of acceptable craniofacial asymmetry has not been clearly defined. The concept of the bilateral symmetry of the human body is also connected with functional symmetry. An assessment of this feature in the craniofacial area is primarily related to the function of the largest and strongest facial bone, namely, the mandible. The symmetrical function of this bone, which is the single and only movable bone of the skull, is determined by two morphologically coupled temporomandibular joints.

In this context, a harmonised relationship between the dental arches is essential for maintaining functional symmetry. Malocclusion, particularly of the transverse type where disrupted symmetry of dental arches can be clinically observed, is a potential cause of functional disorders [3–6]. A priori knowledge clearly indicates the impact of the relationship between dental arches on the function of the masticatory organ [7–10].

The aim of this study was to assess the influence of unilateral posterior crossbite on the electrical activity of the temporal and masseter muscles in patients with subjective symptoms of temporomandibular dysfunctions (TMD).

## 2. Materials and Methods

Fifty patients (22 women and 28 men) aged between 18.4 and 26.3 years (mean 20.84, SD 1.14) with subjective symptoms of TMD (Ai II-III) and unilateral posterior crossbite malocclusion were selected from patients referred to the Pomeranian University in Szczecin, Poland. The control group consisted of 100 subjects (54 women and 46 men) aged between 19.5 and 28.7 years (mean 21.42, SD 1.06) with no malocclusion and subjective symptoms of TMD (Ai I). Patients who had already finished their orthodontic treatment and those who were undergoing treatment at the time of the study were excluded.

The anamnestic interviews included the patients' general medical history as well as detailed information about their masticatory motor system. They were conducted according to a three-point anamnestic index of temporomandibular dysfunction—Ai (Table 1) [11, 12].

The assessment of the function of the masticatory motor system included clinical examination and electromyographic procedures. Clinical examination consisted of visual and auscultatory assessment as well as palpation. This made it possible to accurately and precisely evaluate the function of the masticatory system. Data obtained from the clinical study was analysed using the clinical temporomandibular dysfunction index (Di).

All the patients gave their informed consent to all of the procedures performed.

EMG recordings were performed using a DAB-Bluetooth Instrument (Zebris Medical GmbH, Germany). Each patient was sitting on a comfortable chair without head support and was requested to assume a natural head position.

Surface EMG signals were detected by four silver/silver chloride (Ag/AgCl), disposable, self-adhesive, bipolar electrodes (Noraxon Dual Electrode, Noraxon, USA) with a fixed interelectrode distance of 20 mm. The electrodes were accurately positioned on the anterior temporal muscle and the superficial masseter on both the left and the right sides parallel to the muscular fibres. Anterior temporal muscle is vertically along the anterior margin of the muscle; masseter muscle is parallel to the muscular fibres with the upper pole of the electrode at the intersection between the tragus-labial commissura and exocanthion-gonion lines. A reference electrode was applied inferior and posterior to the right ear [13].

To reduce skin impedance, the skin was cleaned with 70% ethyl alcohol and dried prior to the placement of the electrode. The recordings were performed 5 minutes later.

EMG activity was then recorded during three different tests.

1. Rest activity of the masticatory muscles was performed in the clinical rest position.
2. Maximum voluntary clench (MVC) was performed in the intercuspal position and the subject was asked to clench as hard as possible for 5 seconds.
3. Maximum voluntary clench (MVC) was performed with two 10 mm thick cotton rolls positioned on the mandibular second premolars and molars and the subject was asked to clench as hard as possible for 5 seconds.

To avoid any effects of fatigue, a rest period of at least 5 minutes was allowed between each of the recordings.

For each muscle, the EMG potentials were expressed as a percentage of the MVC value using cotton rolls (unit *μ*V/*μ*V%). This kind of standardization should obviate any variability due to skin and electrode impedance, electrode positioning, and relative muscular hypo- or hypertrophy [14–16].

In the current study, muscular coordination and symmetry of the masticatory muscles were expressed through the use of indices.

The asymmetry between the activity of the left and right jaw muscles was quantified by the Asymmetry Index (As, unit %). This ranges from 0% (total symmetry) to 100% (total asymmetry):

(1) $$\begin{array}{c} \text{As}=\frac{\sum_{i=1}^{N}\left|R_{i}-L_{i}\right|}{\sum_{i=1}^{N}\left(R_{i}+L_{i}\right)}\cdot 100. \end{array}$$

To assess the presence of a possible laterodeviating effect on the mandible during the test caused by unbalanced TR and ML and TL and MR couples, the Torque coefficient (Tc, unit %) was calculated as follows:

(2) $$\begin{array}{c} \text{Tc}=\frac{\sum_{i=1}^{N}\left|{\left(TR+ML\right)}_{i}-{\left(TL+MR\right)}_{i}\right|}{\sum_{i=1}^{N}{\left[\left(TR+ML\right)+\left(TL+MR\right)\right]}_{i}}\cdot 100. \end{array}$$

Tc ranges from 0%, no torque during the test, to 100%, a significant laterodeviating effect on the mandible [17–19].

The Kruskal-Wallis test and the Mann-Whitney *U* test were used to verify the hypotheses relating to the existence or absence of differences between the mean values of the independent variables. The level of significance was set at *P* = 0.05.

The research was approved by the Ethics Committee of the Pomeranian Medical University in Szczecin (number BN-001/45/07).

## 3. Results

**Figure 1:**
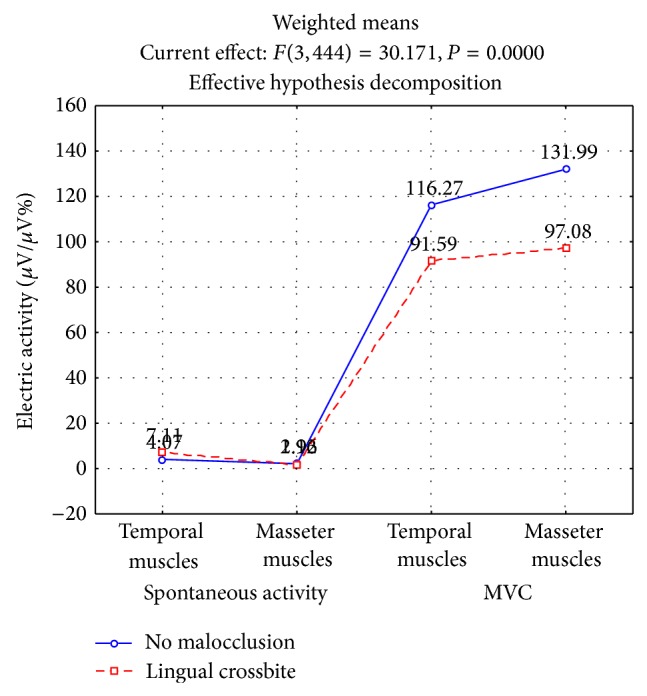
Electrical activity of muscles at clinical mandibular rest position and at maximal voluntary contraction (MVC) in intercuspal position depending on transversal malocclusion.

The analysis of the results of EMG recordings confirmed the influence of unilateral posterior crossbite on the variability of muscle activity in the mandibular rest position (Table 2, Figure 1). The rest activity of the temporal muscles was higher in subjects with crossbite and subjective symptoms of TMD (7.11 *μ*V/*μ*V%, *P* < 0.0249) compared with healthy subjects (4.07 *μ*V/*μ*V%). There were no significant differences in the rest activity of the masseter muscles in either examined group (*P* < 0.5902).

**Figure 2:**
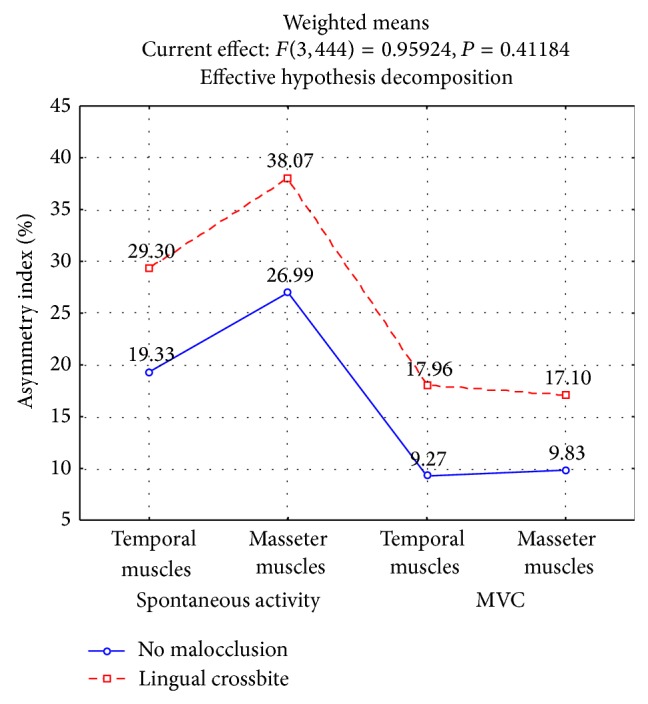
Asymmetry Index (As) of muscles at clinical mandibular rest position and at maximal voluntary contraction (MVC) in intercuspal position depending on transversal malocclusion.

The results showed a significant increase in the Asymmetry Index in relation to both the rest activity of the temporal (29.30%, *P* < 0.0001) and masseter muscles (38.07%, *P* < 0.0006) in patients with unilateral posterior crossbite (Figure 2).

**Figure 3:**
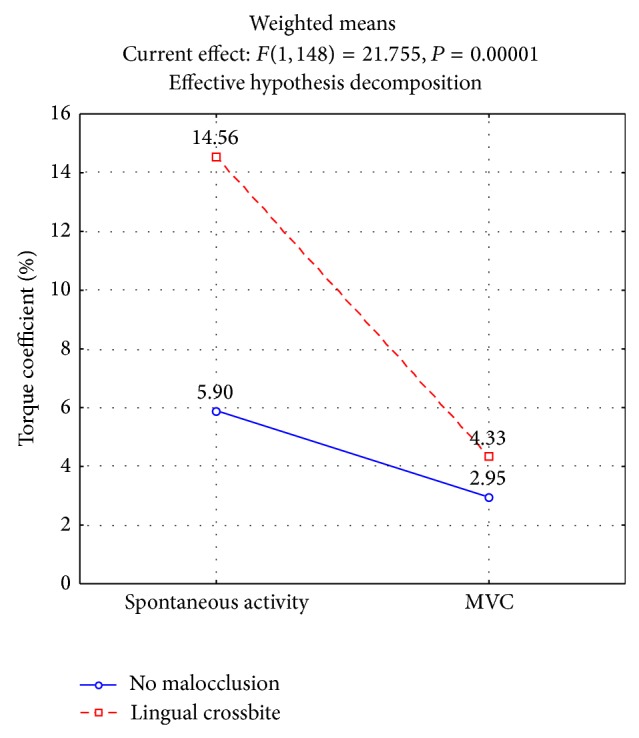
Torque coefficient (Tc) of muscles at clinical mandibular rest position and at maximal voluntary contraction (MVC) in intercuspal position depending on transversal malocclusion.

Additionally, a significant increase was observed in the torque for the pair of muscles responsible for the lateral functional shift of the mandible in the rest position in patients with crossbite (14.56%, *P* < 0.0002, Figure 3).

The differences presented regarding asymmetry in spontaneous muscle activity between the two examined groups were confirmed only for women.

An analysis of the EMG recordings during MVC confirmed the influence of transversal malocclusion on the activity of the masticatory muscles (Table 3, Figure 1). In patients with unilateral posterior crossbite a significant decrease in the activity of the temporal (91.59 *μ*V/*μ*V%, *P* < 0.0000) as well as masseter muscles (97.08 *μ*V/*μ*V%, *P* < 0.0000) in relation to subjects without malocclusion (temporal and masseter muscles 116.27 *μ*V/*μ*V% and 131.99 *μ*V/*μ*V%, resp.) was observed.

The importance of transverse defects was also reflected in significantly higher rates of muscle asymmetry for the temporal (17.96%, *P* < 0.0000) and masseter muscles (17.10%, *P* < 0.0000, Figure 2) during maximum isometric contraction in patients with unilateral posterior crossbite. Analysis revealed a considerable imbalance in the torque for the pair of muscles responsible for the lateral functional shift of the mandible in patients with crossbite (4.33%, *P* < 0.0380, Figure 3).

## 4. Discussion

In our study, an analysis of the influence of the relationships between dental arches on the electrical activity of muscles was performed with respect to two functions: in the mandibular rest position and during maximum isometric contraction. The results confirmed the significant impact of transversal malocclusions on the electrical activity of the temporal and masseter muscles in patients with subjective symptoms of TMD. In the analysis of the results of this scientific experiment, in addition to measurements which were specific for each research method, quotient indicators such as the Asymmetry Index and the Torque coefficient were used. The use of these mathematical tools made it possible to significantly increase the possibilities of describing the biomedical reality. As a result, the unbalanced torque of the two pairs of muscles responsible for the functional lateral shift of the mandible in subjects with unilateral posterior crossbite was observed. This was consistent with the clinical observations. Increased asymmetry in spontaneous muscles activity was revealed only in women and may suggest a higher sensitivity of this examined group for asymmetry of dental arches.

A review of the literature presented by McNamara et al. [20] indicates that there are relatively weak links between function and the alignment of dental arches. Only five occlusal features such as skeletal anterior open bite, overjet greater than 6 to 7 mm, retruded cuspal position/intercuspal position slides greater than 4 mm, unilateral lingual crossbite, and five or more missing posterior teeth have been associated with functional disorders of the masticatory motor system.

Mohlin et al. [21] in a methodical review of 58 studies on the correlations between symptoms of TMD and malocclusions found that there were small differences in terms of functional disorders between subjects with and without malocclusions and thus the authors critically assessed these studies. Moreover, they confirmed a lack of unanimity in the denotation of correlations between function and specific types of malocclusion.

The studies conducted by Egermark-Eriksson et al. [22] in a group of 238 subjects aged 7 to 15 years also showed weak association between functional disorders and malocclusions. The confirmation of this thesis was provided by a lack of variability in the frequency of occurrence for the symptoms of functional disorders in the group who had received orthodontic treatment compared with the group of subjects without such treatment. Nevertheless, with regard to some malocclusions such as crossbite, both uni- and bilateral, anterior open bite, and post- and prenormal occlusion, a higher risk of developing functional disorders was recorded.

Later, a 20-year follow-up by Egermark et al. [23] and Magnusson et al. [24] showed weak correlations between malocclusions and both symptoms and signs of TMD in a group of 402 subjects. Only unilateral crossbite was correlated with symptoms of TMD (*r* = 0.34, *P* < 0.01). Subjects with malocclusion over a long period of time tended to report more subjective symptoms of dysfunctions and to show a higher dysfunction index, compared with subjects with no malocclusion.

An analysis of masticatory muscle activity in patients with altered occlusal relationships due to malocclusion was the subject of studies conducted by Ferrario et al. [25]. The examined group consisted of 10 subjects aged 16–18 years, with posterior unilateral crossbite, bilateral angle Class I, and an overjet and overbite between 2 and 5 mm. The control group consisted of 20 subjects with healthy dentition and with no malocclusion. Electromyographic recordings of masticatory muscles during chewing were performed. The findings of the study showed a decisive influence of crossbite on the electrical activity of the temporal and masseter muscles, manifesting itself in their disturbed coordination. Moreover, the functional changes were more apparent when the side with the altered transversal relationships was directly involved. Similar results were obtained by Rilo et al. [26, 27].

The relationship between transversal malocclusions and the electrical activity of the muscles was described by Alarcón et al. [28]. Electromyographic recordings of anterior and posterior temporal and masseter muscles as well as anterior digastric muscles in 30 subjects with unilateral posterior crossbite and in a control group of 30 normocclusive subjects were made at rest position, during swallowing and during mastication. The results of the study revealed that the posterior temporal muscle on the noncrossbite side was more active than that of the same side in subjects with crossbite at rest position and during swallowing. The activity of both anterior digastrics was higher in subjects with crossbite during swallowing. Moreover, during chewing the masseter muscle was less active in patients with crossbite than in the subjects in the control group. The similar findings were reported by Kecik et al. [29]. The influence of transversal malocclusions on function was confirmed in the prospective electromyographic studies conducted by Sohn et al. [30]. The authors obtained an improvement in masticatory efficiency after orthodontic treatment of anterior crossbite. The duration of muscle activity and the incidence of silent periods in the superficial part of the masseter muscle during chewing in fact decreased after treatment. There were no significant differences in the electrical activity of the anterior and posterior temporal muscles before and after treatment.

Saifuddin et al. [31] assessed the electrical activity of the temporal and masseter muscles in patients with lateral deviations of the mandible (from 5 to 14 mm), crossbite, crowding, and those with no subjective symptoms in the masticatory motor system. The control group consisted of subjects without significant craniofacial asymmetry (acceptable range from 0 to 3 mm), malocclusions, or subjective symptoms of functional disorders. The electromyographic recordings included not just selected activities but also the full daily activity including speech, eating, drinking, and sleeping. The analysis of muscle activity was divided into three periods: ordinary daily activities, mealtimes, and sleeping. The results showed that muscle activity in patients with disorders was significantly lower during all three periods for the masseter muscle and during ordinary daytime activities for the temporal muscle, in comparison to the control group. The Asymmetry Index (AI) in patients with a lateral shift of the mandible was significantly greater during usual daytime activities and sleep for the temporal muscle and significantly smaller during sleep for the masseter muscle, in comparison to the control group. The results clearly revealed that the asymmetry in the electrical activity during ordinary daytime activities and sleep in patients with lateral deviations of the mandible to a greater extent affects temporal muscles (anterior part) than masseter muscles. According to the authors, a reduction in temporal and masseter muscle electrical activity, with the accompanying asymmetry in the electrical activity of the temporal muscles, is closely related to occlusal instability due to malocclusions and lateral mandibular deviation.

A review of the literature presented does not indicate a clear association between malocclusions and TMD. However, the results of the aforementioned studies suggest a higher risk of the prevalence of TMD in patients with unilateral posterior crossbite.

## 5. Conclusions

The use of sEMG in the assessment of the function of the masticatory motor system provided tangible evidence of the determining influence of unilateral posterior crossbite on the electrical activity of the temporal and masseter muscles in patients with subjective symptoms of TMD.

## Figures and Tables

**Table 1:** Anamnestic index of temporomandibular dysfunction (Ai).

Ai	Symptoms
I	No subjective symptoms of temporomandibular dysfunction: no symptoms reported by patient.

II	Mild symptoms of temporomandibular dysfunction: temporomandibular joint noise, feeling of “jaw fatigue” (fatigue of masticatory muscles), and feeling of “jaw rigidity” (increased tone of masticatory muscles).

III	Severe symptoms of temporomandibular dysfunction: restricted mouth opening, painful lower jaw movements, temporomandibular joint pain, masticatory muscle pain, temporomandibular joint luxation, and lockjaw.

**Table 2:** Electrical activity of muscles at clinical mandibular rest position depending on transversal malocclusion.

Region	Variable	Gender	Group					
			No malocclusion			Crossbite		
			*n*	Mean	SD	*n*	Mean	SD
Temporal muscles	Electrical activity [*μ*V/*μ*V%]	Females	54	4.07	2.02	22	7.08	6.45
		Males	46	4.08	2.06	28	7.14	5.10
		Total	100	4.07	2.03	50	7.11	5.67
	Asymmetry index [%]	Females	54	21.20	9.48	22	36.35	12.71
		Males	46	17.12	9.00	28	23.77	15.60
		Total	100	19.33	9.44	50	29.30	15.59

Masseter muscles	Electrical activity [*μ*V/*μ*V%]	Females	54	2.12	0.89	22	1.78	0.98
		Males	46	2.13	1.09	28	2.10	0.57
		Total	100	2.12	0.98	50	1.96	0.79
	Asymmetry index [%]	Females	54	23.03	14.97	22	45.78	19.99
		Males	46	31.63	14.14	28	32.01	17.37
		Total	100	26.99	15.15	50	38.07	19.63

Temporal/masseter muscles	Torque coefficient [%]	Females	54	7.20	7.66	22	19.43	13.53
		Males	46	4.37	3.95	28	10.73	12.83
		Total	100	5.90	6.36	50	14.56	13.72

**Table 3:** Electrical activity of muscles at maximal voluntary contraction (MVC) in intercuspal position depending on transversal malocclusion.

Region	Variable	Gender	Group					
			No malocclusion			Crossbite		
			*n*	Mean	SD	*n*	Mean	SD
Temporal muscles	Electrical activity [*μ*V/*μ*V%]	Females	54	117.86	28.11	22	82.88	19.33
		Males	46	114.40	27.06	28	98.43	34.86
		Total	100	116.27	27.54	50	91.59	29.84
	Asymmetry index [%]	Females	54	9.95	8.18	22	22.10	11.14
		Males	46	8.48	5.73	28	14.71	9.81
		Total	100	9.27	7.16	50	17.96	10.95

Masseter muscles	Electrical activity [*μ*V/*μ*V%]	Females	54	131.06	26.23	22	76.72	37.22
		Males	46	133.07	33.94	28	113.08	42.73
		Total	100	131.99	29.89	50	97.08	43.96
	Asymmetry index [%]	Females	54	9.07	5.23	22	21.72	12.81
		Males	46	10.71	6.18	28	13.48	5.01
		Total	100	9.83	5.72	50	17.10	10.06

Temporal/masseter muscles	Torque coefficient [%]	Females	54	2.77	1.88	22	6.62	4.44
		Males	46	3.16	2.35	28	2.54	1.49
		Total	100	2.95	2.11	50	4.33	3.72
